# Bridging the Gap in Chronic Disease Management: A Nursing Perspective on the Use of Predictive Tools and Telemedicine in the Hospital–Community Transition

**DOI:** 10.3390/medicina61122213

**Published:** 2025-12-15

**Authors:** Gianluca Azzellino, Mauro Passamonti, Ernesto Aitella, Luca Mengoli, Patrizia Vagnarelli, Lia Ginaldi, Massimo De Martinis

**Affiliations:** 1 Department of Life, Health and Environmental Sciences, University of L’Aquila, 67100 L’Aquila, Italy; 2 Complex Operational Unit, Adriatic District Area, AUSL 04 Teramo, 64100 Teramo, Italypatrizia.vagnarelli@aslteramo.it (P.V.); 3 Allergy and Clinical Immunology Unit, Center for the Diagnosis and Treatment of Osteoporosis, AUSL 04 Teramo, 64100 Teramo, Italy; 4 Long-Term Care Unit, “Maria SS. dello Splendore” Hospital, AUSL 04 Teramo, 64021 Giulianova, Italy; 5 UniCamillus—Saint Camillus International University of Health Sciences, 00131 Rome, Italy

**Keywords:** chronic disease, continuity of care, patient discharge, telemedicine, hospital readmission, nurse case managers, family nurse

## Abstract

Chronic diseases represent one of the most complex, costly, and significant challenges for healthcare systems. The increase in chronic conditions and multimorbidity, together with the growing demand for continuity of care makes the vulnerability of the hospital-to-community transition increasingly evident. This phase is often characterized by delays, fragmented services, and insufficient support for patients and caregivers, leading to higher rates of early readmission and substantial clinical, social, and economic impacts. This paper was developed through a narrative synthesis of international and national literature on continuity of care, integrated models, and nurse-led experiences. Based on this synthesis, an integrated six-phase nursing model is proposed, combining predictive assessment tools and telemedicine to enhance early risk identification, proactive discharge planning, and post-discharge follow-up. Evidence indicates that nurse-led interventions supported by digital solutions can reduce inappropriate hospital days, decrease hospital readmissions, and improve patient and caregiver satisfaction. The integration of predictive tools and telemedicine solutions, coordinated by nurse case managers, represents a promising strategy to strengthen continuity of care and the sustainability of the healthcare system, and the proposed conceptual model highlights practical implications while outlining future research directions for empirical validation and large-scale implementation.

## 1. Introduction

Chronic conditions represent one of the most complex and costly challenges for healthcare systems worldwide. Recent European data show that over 40% of adults aged 65 and over live with two or more chronic diseases, and that more than half of the years lived after age 65 are affected by chronic conditions or disabilities, increasing the need for long-term and integrated care [1]. In Italy, about 40% of the population has at least one chronic disease and over 20% has two or more, with prevalence exceeding 70% among people aged 75 and over [2]. In this context, Ministerial Decree 77/2022 and the National Recovery and Resilience Plan (PNRR—Mission 6 Health) define a new territorial care model based on hospital–community integration and on the introduction of roles such as the Family and Community Nurse, with the aim of reducing fragmentation and strengthening continuity and equity in chronic care [3]. Population ageing and rising multimorbidity demand a reorganization of healthcare systems toward proximity, integration and continuity supported by multidimensional and multidisciplinary care models. These reforms aim to overcome the fragmentation of care pathways and make health systems more proactive, equitable, and sustainable in addressing chronicity [3].

The transition from hospital to community care is a particularly vulnerable phase, often characterized by discontinuity and fragmentation, with negative outcomes for patients and caregivers. Hansen et al. (2011) found that inadequate discharge documentation and the absence of risk stratification are associated with an increased risk of hospital readmission [4]. Randomized controlled trials conducted by Naylor et al. (2004) and Coleman et al. (2006) demonstrated that nurse-led transitional care programs significantly improve outcomes in patients with chronic conditions such as heart failure [5,6]. Gao et al. (2022), Vinci et al. (2024), and Binda et al. (2025) highlighted that non-clinical barriers, such as organizational inefficiencies, are among the main determinants of delayed discharges [7,8,9]. Bechir and Bechir (2025) and Azzellino et al. (2025) reported that the introduction of roles dedicated to discharge coordination, such as the nurse case manager, can reduce delays and improve care flow [10,11].

However, patient caregivers are often excluded from decision-making processes, receiving limited support and training, factors that contribute to early readmissions and reduce quality of life [5,12,13]. In addition, post-discharge follow-up is often neglected, with limited outcome verification and clinical monitoring [6]. The integration of telemedicine and telemonitoring programs has been shown to improve satisfaction and reduce avoidable hospitalizations [14,15].

At the same time, the introduction of Family and Community Nurses has been promoted to strengthen hospital–community continuity, although heterogeneity in models and outcome indicators persists [11,16,17]. Transitional care refers to time-limited interventions aimed at ensuring continuity and coordination during transfers between care settings through proactive discharge planning, patient and caregiver education, and structured post-discharge follow-up, particularly for individuals with chronic and complex conditions [5,6]. The concept of integrated care, as defined by the World Health Organization in the Framework on Integrated People-Centred Health Services [18], emphasizes the coordination of services across levels and sectors to address patients’ multidimensional needs and reduce system fragmentation. Established transitional care models—including the Naylor Transitional Care Model and the Coleman Care Transitions Intervention—demonstrate the effectiveness of nurse-led coordination, education, and follow-up in improving continuity of care [5,6]. In Italy, recent experiences reported by Azzellino et al. (2025) and Scrimaglia et al. (2024) highlight the potential of Family and Community Nurse–based approaches to support integrated hospital-to-community transitions [16,19].

The nursing perspective is naturally aligned with these principles, as nursing models are intrinsically person-centered and based on continuity, proximity, coordination, and education. Within the OECD framework for strengthening primary and community care, nurses play a key role in bridging hospital and home, ensuring relational and informational continuity and enabling early activation of community services [1]. Theories of continuity of care distinguish between informational, management, and relational continuity, all of which are essential to ensure smooth and safe transitions and to improve the quality of care. Italy is progressively implementing these principles through Ministerial Decree 77/2022 [3], which promotes a new organizational paradigm based on proximity, multidisciplinarity, and integrated care, led by the Family and Community Nurse [20,21].

Predictive models are increasingly used in healthcare to estimate the risk of hospital readmission by integrating clinical, functional, and sociodemographic variables. These tools support clinicians in identifying patients who may require more intensive discharge planning or closer follow-up after hospitalization [22]. In parallel, telemedicine has emerged as a key component of modern transitional care, offering remote monitoring, timely detection of clinical deterioration, and extended support for patients once they return home. Recent evidence shows that telemonitoring interventions can help reduce hospital readmissions and strengthen continuity of care for chronic patients [14], a finding consistent with earlier landmark trials in the field of telehealth [15].

Building on these theoretical frameworks, this paper examines how predictive tools and telemedicine can support nursing-led transitional care models in Italy. Despite growing interest in integrated care, significant gaps remain in the evidence on hospital-to-community transitions, particularly regarding nurse-led coordination, predictive assessment, and the impact of the nurse case manager in chronic and post-acute settings. To address these gaps, we conducted a narrative synthesis of the literature on transitional care, discharge planning, and integrated nursing models, complemented by insights from real-world Italian experiences. The conceptual model was developed through a targeted and iterative review of studies on transitional care, nurse case management, predictive tools, and telemedicine. Key mechanisms influencing continuity, such as early risk identification, discharge coordination, caregiver involvement, and telehealth-supported follow-up, were extracted and organized into thematic domains. These domains were progressively compared and refined, leading to the formulation of the proposed six-phase model. The resulting framework integrates predictive analytics and telemedicine to enhance coordination, continuity, and sustainability across the hospital–community interface, offering both a theoretical foundation and operational guidance for future implementation [12,16,23,24].

However, the literature consistently highlights structural limitations that continue to undermine the effectiveness of transitional care. Organizational fragmentation hampers early risk identification and the development of individualized discharge pathways [5,12,13]; persistent gaps in caregiver support remain; and follow-up practices, despite strong evidence of benefit, vary widely across settings [6,17,25]. These weaknesses contribute to avoidable readmissions, increased costs, and reduced quality of life, and are largely attributable to insufficient caregiver involvement and the absence of structured follow-up strategies [5,6,12,13,23].

## 2. Why the Role of the Nurse Case Manager Is Important?

Case management is a collaborative and patient-centered process that involves assessment, planning, coordination, and evaluation of care across different settings to promote continuity, appropriateness, and quality of healthcare delivery [26]. Within this framework, the nurse case manager acts as a coordinating professional who integrates clinical, organizational, and communication competencies to ensure continuity, safety, and efficiency during the hospital-to-community transition [27]. In many healthcare systems, case management nurses possess advanced or postgraduate education, often at master’s level or through specialized training in care coordination and complex discharge management, corresponding to advanced practice nursing standards [26,28]. Evidence from different international contexts indicates that nurse case managers share a core set of competencies including multidisciplinary coordination, patient and caregiver education, and the ability to navigate complex health systems [26,29]. However, relevant differences exist in the operational models adopted across countries, reflecting variability in organizational structures, professional regulation, and local training pathways [27,30].

The nurse case manager represents a key figure in ensuring continuity of care, thanks to the ability to integrate clinical, organizational, and communication skills. Building on these competencies, several core functions characterize effective nurse-led transitional care, including early risk assessment, structured coordination, and caregiver support. Early assessment of frailty and risk of complex discharge is essential, as already during hospital admission the nurse can identify patients at risk of complex discharge through the use of validated scales. Studies show that early assessment is essential to prevent delays and reduce the phenomenon of bed blocking [31]. Similarly, studies show that the absence of a structured assessment leads to discharge delays and consequently to prolonged hospital stays [7]. Analyses of recent studies have quantified the impact of delayed discharges, showing a significant consumption of hospital resources and negative consequences on care pathways [8,9].

Coordination between hospital and community services is another central component of the nurse case manager’s role. The work of the nurse case manager serves as a bridge between hospital and community care. One study demonstrated that the presence of a dedicated coordination role significantly reduces discharge delays and improves patient flow [10]. Moreover, a scoping review showed that the role of the case manager is essential to ensure integration across care settings [11].

Education and support for patients and caregivers also represent a core function. One of the activities of the nurse case manager is caregiver empowerment. Bhandari et al. (2022) reported that structured transitional programs including counseling and support increase therapeutic adherence [12]. Similarly, Everall et al. (2019) found that the absence of caregiver support is one of the main causes of early readmission and deterioration in quality of life [13].

Integration of digital solutions and telemonitoring is increasingly relevant. The nurse case manager is the most suitable professional to integrate telehealth solutions into discharge pathways. Elsener et al. demonstrate that the use of telemonitoring, managed by nursing staff, reduces readmissions and increases patient satisfaction [23]. Supporting this hypothesis, a systematic review confirms the effectiveness of nurse-led interventions supported by digital technologies [32].

Finally, reduction in readmissions and improvement of sustainability are key outcomes associated with this role. Naylor et al. (2004) and Azzellino et al. (2025) demonstrated that nursing interventions activated during protected hospital-to-community discharge, combined with post-discharge follow-up, reduce the risk of hospital readmission [5,16]. Specifically, these authors reported that the application of integrated models coordinated by a nurse case manager leads to a significant reduction in 30-day readmissions and an improvement in patient and caregiver satisfaction [5,16]. Within this framework, digital innovation and telehealth represent essential enablers of nurse-led transitional care, extending continuity and monitoring beyond hospital walls.

## 3. Proposed Nursing-Led Transitional Care Model

Tyler et al. (2023) reported that the most effective hospital-to-community transition models are nurse-led and based on systemic and multidimensional approaches [33]. Evidence indicates the need to implement structured, multi-phase pathways that include early assessment, anticipatory planning, coordination, education, and digital follow-up [4,6,23]. An integrated nursing transition model for frail or chronic patients can be structured into six phases. The proposed model integrates predictive assessment and telemonitoring as cross-cutting elements to ensure early identification of risks and sustained follow-up after discharge.

1. 1. Early screening using predictive scores.

Early identification of patients at risk of complex discharge is essential to ensure timely planning and prevent unplanned readmissions. Tools such as the Blaylock Risk Assessment Screening Score (BRASS) [34] have demonstrated good predictive validity for identifying patients at high risk of prolonged hospitalization or complex discharge. Colognesi et al. (2021) confirmed its usefulness in surgical departments, emphasizing its value in the early identification of patients requiring complex post-hospital care, while Louis Simonet et al. (2008) demonstrated that the BRASS effectively predicts discharge to post-acute facilities and supports early discharge planning [35,36]. Similarly, Azzellino et al. (2025) showed that the early use of structured assessment tools such as the BRASS, integrated within protected discharge and case management pathways, contributes to a significant reduction in hospital readmissions [16]. Building on these experiences, predictive approaches based on electronic health records and artificial intelligence are emerging as promising tools to enhance discharge coordination and continuity of care [22,37].

2. Anticipatory planning with a target discharge date.

Bechir and Bechir (2025), Henry et al. (2021), and Abuzied et al. (2021) found that introducing an estimated discharge date reduces unnecessary hospital days and improves patient flow [10,38,39,40]. The case manager nurse, involved from the very first days, facilitates multidisciplinary planning and the timely activation of community services.

3. Early activation of community home care, Long-Term Care Facilities (LTCFs), and social support.

Non-clinical barriers (limited bed availability, bureaucratic difficulties, administrative delays) are among the major causes of delayed discharge [41,42]. Vinci et al. (2024), Hernández et al. (2018), and Lin et al. (2024) reported that innovative models enabling early activation of community home care (within 24–48 h) reduce hospital length of stay and promote continuity of care [8,43,44].

4. Caregiver education and the teach-back method

Training caregivers is a fundamental step in the hospital-to-community transition. Bhandari et al. demonstrated that educational programs based on the teach-back method increase treatment adherence while simultaneously reducing rehospitalization [12,45,46]. Azzellino et al. (2025) confirmed the importance of the case manager’s educational role in including the caregiver in the care plan [11].

5. Telehealth follow-up

Telemonitoring and digital follow-up are cited in the literature as interventions with a positive impact on post-discharge outcome [14,15]. Elsener et al. focused their study on the combined use of vital signs telemonitoring and digital telephone follow-up, demonstrating a reduction in the rate of early rehospitalization [23]. Evidence from established transitional care models shows that early nurse contact within 48–72 h after discharge, followed by structured follow-up during the first two weeks, represents a critical window to detect early signs of clinical instability and prevent avoidable readmissions [5,6].

6. Outcome assessment

Key indicators include: inappropriate hospital days, time to activation of home care services, 30-day readmissions, and caregiver satisfaction. A scoping review highlights that systematic outcome monitoring improves perceived quality and reduces the psychological impact of difficult discharges [13].

**Figure 1:**
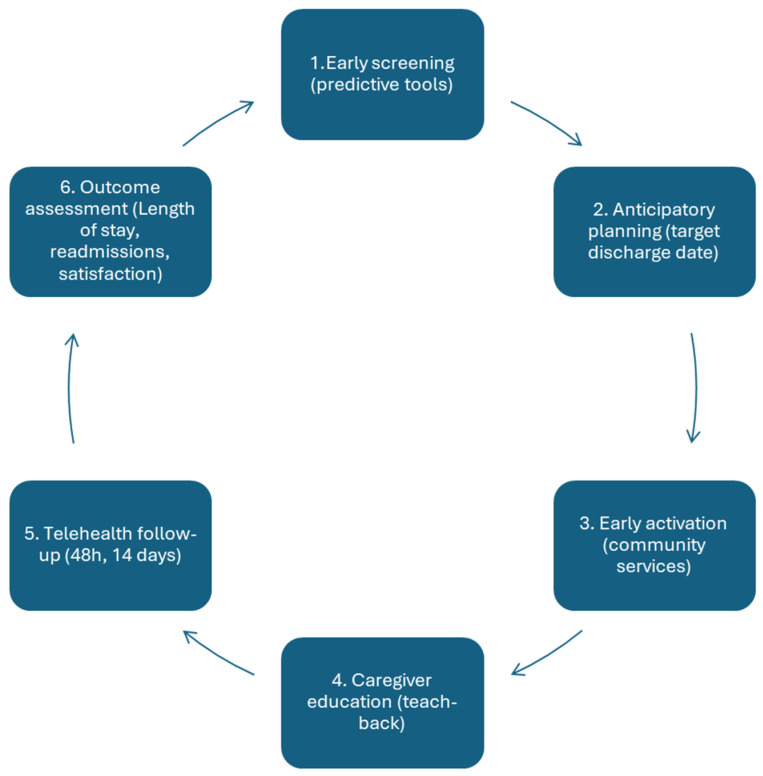
Proposed Six-Phase Nursing-Led Transitional Care Model.

Although this six-phase model has not yet been empirically validated as an integrated framework, each phase is supported by evidence from transitional care research. Studies indicate that early risk assessment, anticipatory discharge planning, timely activation of community services, caregiver education, telehealth follow-up, and outcome monitoring improve continuity of care and reduce hospital readmissions [5,12,16,33,36]. The model is therefore conceptual, grounded in existing evidence and policy frameworks, and requires future validation through implementation studies. Figure 1 illustrates the six interconnected phases of the hospital-to-community transition cycle.

## 4. Implications for Practice and Policy

Evidence from recent systematic reviews and meta-analyses demonstrates that nurse-led transitional care models are among the most effective strategies to improve continuity between hospital and community care. These interventions—characterized by multidimensional coordination, early assessment, and structured follow-up—significantly reduce hospital readmissions and enhance patient outcomes [25,33,47,48,49]. Building on this evidence, the following actions are required to consolidate integrated care models:

1. Formally include the nurse case manager in hospital-to-community care pathways (PDTA).

The nurse case manager and the discharge coordinator play distinct yet complementary roles within the hospital-to-community transition process. The discharge coordinator primarily manages the organizational and logistical aspects of discharge planning, facilitating timely patient flow and communication with community services. Recent evidence confirms that the introduction of a dedicated discharge coordinator significantly reduces discharge delays and improves patient throughput [10,20]. Conversely, the nurse case manager operates within a broader clinical and organizational framework, overseeing the entire continuum of care, from early assessment and anticipatory planning to post-discharge follow-up, to ensure continuity, quality, and coordination across settings. Studies have highlighted that nurse case managers contribute to improved integration between hospital and community services and play a central role in reducing rehospitalizations [16,27,30]. The integration of *hospital-to-community care pathways (PDTA)* within discharge and transitional care processes, is essential to ensure coordinated and person-centred care. Recent evidence and institutional frameworks highlight that embedding nurse case managers within these integrated pathways facilitates early activation of community services, improves continuity, and reduces fragmentation across care settings [50].

2. Invest in digital competencies and advanced nursing training

The integration of telemedicine into transition phases requires new skills. Studies show that nurse-led telehealth models reduce early rehospitalizations [23,51]. This implies the introduction of specific training programs in digital health, case management, and caregiver communication.

3. Ensure interoperability of data between hospital and community care

One major barrier identified in the literature is poor communication between hospital and community services, often linked to fragmented information flows and organizational barriers [7]. The lack of interoperable platforms slows down communication and makes the early activation of community care services ineffective. Investing in integrated digital systems is essential to operationalize an effective transition model.

4. Measure outcomes with monitoring dashboards.

Indicators such as length of stay, inappropriate hospital days, 30-day readmission rates, and time to activation of community care have been used in several studies [8,9,41]. Systematic monitoring with clinical-organizational dashboards not only enables the measurement of intervention impact but also allows for the timely identification of critical issues in transition processes.

## 5. Discussion

This paper examined how predictive tools and telemedicine can enhance the hospital-to-community transition for patients with chronic conditions through a nurse-led, structured, and evidence-based model. A large body of literature shows that nurse-led transitional care interventions consistently reduce hospital readmissions and strengthen continuity of care by integrating early assessment, education, and follow-up [5,6,33,48,49]. Building on these insights, the proposed six-phase model offers a proactive pathway in which predictive analytics and telehealth play complementary roles. Predictive tools constitute the foundation of early risk identification and individualized discharge planning. Traditional instruments such as the BRASS, Barthel Index, and ADL/IADL scales evaluate functional, cognitive, and social domains that are known to influence discharge outcomes [34,35,36,52,53]. However, these tools alone cannot fully capture the multidimensional determinants of readmission risk. Recent studies show a progressive shift toward more advanced predictive models that integrate heterogeneous data—including electronic health records, administrative data flows, wearable sensors, and patient-reported information collected through apps and digital platforms [54,55,56,57,58,59]. Such models offer more comprehensive assessments and support tailored interventions, although methodological heterogeneity, limited inclusion of nursing-sensitive indicators, and lack of real-world validation still constrain their application in practice. Telemedicine extends this predictive capacity into the post-discharge period by providing continuous monitoring, timely detection of deterioration, and structured follow-up. Nurse-led telehealth programs that combine telemonitoring with scheduled video or telephone consultations have been shown to reduce 30-day readmissions and improve continuity of care and patient satisfaction [15,23,25]. Prospective studies confirm that integrating telehealth into transitional pathways facilitates communication between hospital and community services, enhances caregiver engagement, and improves adherence to care plans [12,33,47]. Despite these benefits, challenges persist: many studies involve small samples or short follow-up periods, outcome reporting remains inconsistent, and interoperability between hospital and community digital systems is still limited [60]. Organizational and technological barriers further contribute to uneven implementation across healthcare settings.Taken together, the evidence indicates that combining predictive analytics with telemedicine enables a more proactive, personalized, and continuous transition process. Predictive tools identify high-risk patients early, while telemonitoring supports longitudinal oversight after discharge. These elements converge within the proposed six-phase model, which integrates early screening, anticipatory planning, timely activation of community services, caregiver education, digital follow-up, and systematic outcome assessment. By embodying the principles of continuity, integration, and person-centeredness, the model offers a feasible pathway to closing existing gaps in transitional care and supporting ongoing healthcare transformations at national and European levels.

## 6. Conclusions

The integration of predictive tools and telemedicine solutions, led by nurse case managers, represents an innovative and evidence-based strategic element for managing chronic conditions during the hospital–community transition. This approach helps reduce hospital readmission rates and discharge delays, improves patient and caregiver satisfaction, and contributes to the long-term sustainability of healthcare systems. Beyond clinical outcomes, this model emphasizes the strategic role of nurse leadership in driving digital innovation and coordinating multidisciplinary care pathways. Investing in continuous education, digital competence, and the development of advanced nursing skills is essential to ensure the safe, ethical, and effective implementation of predictive and telehealth technologies in clinical practice. Ultimately, redefining the hospital–community transition through a nurse-led, predictive, and digital paradigm has the potential to transform not only the quality and continuity of care, but also the sustainability and equity of chronic care management across European health systems.

## 7. Future Directions and Research Agenda

Despite promising evidence, significant research gaps persist. Most studies are single-center or exploratory, with small samples and limited follow-up, reducing generalizability. Research in post-acute and community settings remains scarce, and outcome reporting is heterogeneous, with little standardization of indicators such as length of stay, readmissions, caregiver satisfaction, and patient-reported outcomes. The economic evaluation and sustainability of digital, nurse-led models are also rarely addressed, and few studies have analyzed the contextual factors influencing their implementation in daily practice. Future research should prioritize large-scale, real-world evaluation of integrated nurse-led transitional care models, supported by predictive analytics and telemedicine to enhance early risk identification, continuity, and patient engagement. The next step is to empirically validate the proposed six-phase conceptual model through multicenter implementation studies assessing its impact on readmissions, caregiver satisfaction, and continuity of care indicators. Multicenter and longitudinal designs should adopt composite outcomes, including clinical, experiential, and economic measures, to capture the full impact of such models. Finally, the integration of artificial intelligence and predictive tools for personalized discharge planning represents a promising frontier for strengthening continuity, safety, and efficiency in the hospital–community transition.

## Data Availability

All data generated or analyzed during this study are included in this article.
